# Effects of Water Loss Stress under Tidal Effects on the Epiphytic Bacterial Community of *Sargassum thunbergii* in the Intertidal Zone

**DOI:** 10.1128/msphere.00307-22

**Published:** 2022-09-29

**Authors:** Tao Sun, Zhibo Yang, Jun Chen, Yang Li, Jing Wang, Xiya Wang, Xuexi Tang, Hui Xiao

**Affiliations:** a College of Marine Life Sciences, Ocean University of China, Qingdao, China; b Laboratory for Marine Ecology and Environmental Science, Qingdao National Laboratory for Marine Science and Technology, Qingdao, China; University of Michigan-Ann Arbor

**Keywords:** epiphytic bacterial community, water loss stress, 16S rRNA high-throughput sequencing, *Sargassum thunbergii*

## Abstract

Intertidal macroalgae face periodic water loss and rehydration caused by daily tidal changes. However, the effect of water loss stress on algal epiphytic bacteria has not yet been reported. In this study, the effects of water loss stress on the epiphytic bacteria community of *Sargassum thunbergii* were analyzed, and the different responses of epiphytic bacteria to water loss stress were compared between male and female algae. The results showed that after water loss stress, the diversity of the epiphytic bacterial community of *S. thunbergii* first decreased and then increased. Among the dominant taxa, the abundance of *Cyanobacteria* decreased significantly, whereas the abundance of *Portibacter* and *Aquimarina* first increased and then decreased. Additionally, the indicator species and the abundance of predicted functional genes related to carbon, nitrogen, and sulfur metabolism both changed significantly. More importantly, when the epiphytic bacteria were analyzed separately according to the algal sex, the changes in algal epiphytic bacterial community structure and indicator species were more significant, and there were sexual differences. Therefore, it was concluded that water loss stress has a significant effect on the community structure and function of the epiphytic bacteria on *S. thunbergii*. Meanwhile, the epiphytic bacteria community of two sexes of *S. thunbergii* differed in the response to water loss stress.

**IMPORTANCE** Periodic water loss caused by the tide is an important environmental factor that is faced by intertidal macroalgae, but the impact of periodic water loss on the epiphytic bacterial communities associated with macroalgae is still unknown. Through this study, we found that the diversity, the relative abundance of dominant taxa, the indicator species, and the abundance of the predicted functional genes in the epiphytic bacteria on *S. thunbergii* changed with the time of water loss. Moreover, male and female *S. thunbergii* exhibited different responses to water loss stress. This study not only paves the way for the delineation of the interactions between *S. thunbergii* and its epiphytic bacteria but also provides new insights for the mechanisms of the adaptation and evolution of macroalgae in the intertidal zone.

## INTRODUCTION

Drought and water loss occur frequently, all over the world, and are among the most unfavorable abiotic stress factors in the process of plant growth and development (1, 2). Intertidal macroalgae play a crucial role in the marine ecosystem, not only providing protection and food sources for a variety of organisms but also participating in the construction of habitats for many species in the intertidal zone (3, 4). Due to the unique ecological environment at the land-sea junction of the intertidal zone, the macroalgae living there experience periodic water loss and rehydration cycles (5). Therefore, the effect of water loss stress on intertidal algae has become a hot topic for many scholars. Numerous studies have previously reported the effects of water loss stress on a variety of intertidal algae (including members of the red, green, and brown algal phyla). The results showed that water loss stress leads to the contraction of protoplasts, the disintegration of thylakoids to different degrees, a decrease in photosynthetic activity, an increase in active oxygen content, an increase in tolerance-related proteins, and the significant expression of antioxidant enzyme genes (6–10).

On the surfaces of algae are epiphytic bacterial symbionts, which play an indispensable role in the life history of algae (11, 12). Algae without epiphytic bacteria cannot maintain their normal growth and physiological activities (13–15). At low tide, the epiphytic bacteria and the algae are inevitably subjected to water loss stress at the same time. Previous studies have shown that microbiota can help host plants improve their tolerance to drought stress (16). In turn, host plants can also help these organisms resist drought stress by regulating the responses of the microorganisms (17, 18). Clarifying the responses of the plant epiphytic bacterial community to changes in environmental factors can provide a better understanding of the mechanisms of bacteria-algae interactions under environmental stress. At present, a variety of environmental factors, including nitrogen and phosphorus nutrients (3), copper pollution (19), temperature (20), and salinity (21), have been proven to have significant effects on the algae epiphytic bacterial community, including changes to the community structure, biodiversity, and the relative abundance of dominant taxa, but the response of the macroalgae epiphytic bacterial community structure to water loss stress has not yet been reported.

In addition, studies have shown that dioecious plants have significant morphological and physiological differences in response to drought stress. Zhang et al. (22) pointed out that under drought stress, male Populus cathayana have a stronger antioxidant capacity, osmotic pressure regulating capacity and photosynthesis protection capacity than do females. At the same time, the sex differences of *Populus cathayana* under drought stress were related to the differential expression of sex-dependent proteins (23). There are also reports that further indicate that the expression levels of genes related to photosynthesis and active oxygen scavenging enzymes in male Populus yunnanensis were significantly higher than those observed in female *Populus yunnanensis* (24). Lin et al. (25) reported that during the dehydration process of Porphyra katadai var. *hemiphylla* growing in the intertidal zone, the photosynthetic system I of females was damaged more than that of males as well as that the male photosynthetic system I could restore photosynthesis after rehydration, while the female system could not. The above studies showed that the effects of drought and water loss stress on plants display sex-based differences. The relationships between plants, including macroalgae, and their epiphytic surface bacteria are complex, and there are close and active relationships between these organisms (26). When drought or water loss stress occurs, the physiological changes of host plants inevitably leads to a corresponding response and adjustment of the epiphytic bacterial community, and this response and adjustment are closely related to the metabolic activities of the plant. When studying the responses of epiphytic bacteria to stress, because the sexual differences of host plants in response to stress and other changes can even be opposite (27), the changes in the community may be confused or even offset, making it impossible to accurately judge the effects of stress factors on the epiphytic bacteria of host plants. Therefore, it was necessary to separately analyze the effects of stress factors on the epiphytic bacterial community according to the sex of the host plants to more accurately explain the mechanisms by which stress affects the relationships between bacteria and algae.

Sargassum thunbergii is a common intertidal dioecious macroalga in the northern sea area of China, a dominant habitat-forming species of kelp beds, and an important bait for mariculture (28). Based on 16S rRNA high-throughput sequencing, this study elucidates the differences in epiphytic bacterial community structure and function in intertidal *S. thunbergii* under different durations of water loss and further compares the differential responses of male and female alga epiphytic bacterial communities to water loss stress in order to reveal the mechanisms of interactions between algae and epiphytic bacteria under water loss conditions. Then, this study also provides an experimental basis for the protection and utilization of *S. thunbergii*.

## RESULTS

This study included 24 samples, and a total of 1,920,675 pairs of reads were obtained, with 1,914,021 clean reads generated after quality control and splicing. The removal of chimeras yielded an average of 73,860 effective reads per sample (Table S1). Sequence clustering was performed at a 97% similarity level, and a total of 1,942 bacterial operational taxonomic units (OTUs) were obtained from 24 samples, of which male samples contained 1,941 OTUs and female samples contained 1,942 OTUs. The bacterial community coverage of all samples was greater than 99%. The rarefaction curve and rank abundance curve showed that the sequencing depth was sufficient to describe the bacterial richness and diversity in all samples (Fig. S1).

10.1128/msphere.00307-22.1 FIG S1 (A) Rarefaction curve analysis of all samples. (B) Rank abundance curve analysis of all samples. Download FIG S1, TIF file, 1.0 MB. Copyright © 2022 Sun et al. 2022 Sun et al. https://creativecommons.org/licenses/by/4.0/ This content is distributed under the terms of the Creative Commons Attribution 4.0 International license.

10.1128/msphere.00307-22.2 TABLE S1 Sequencing data of the epiphytic bacterial community of *S. thunbergii*. Download Table S1, DOCX file, 0.01 MB. Copyright © 2022 Sun et al. 2022 Sun et al. https://creativecommons.org/licenses/by/4.0/ This content is distributed under the terms of the Creative Commons Attribution 4.0 International license.

### Effect of water loss stress on the α-diversity of the epiphytic bacterial community of *S. thunbergii*.

**FIG 1:**
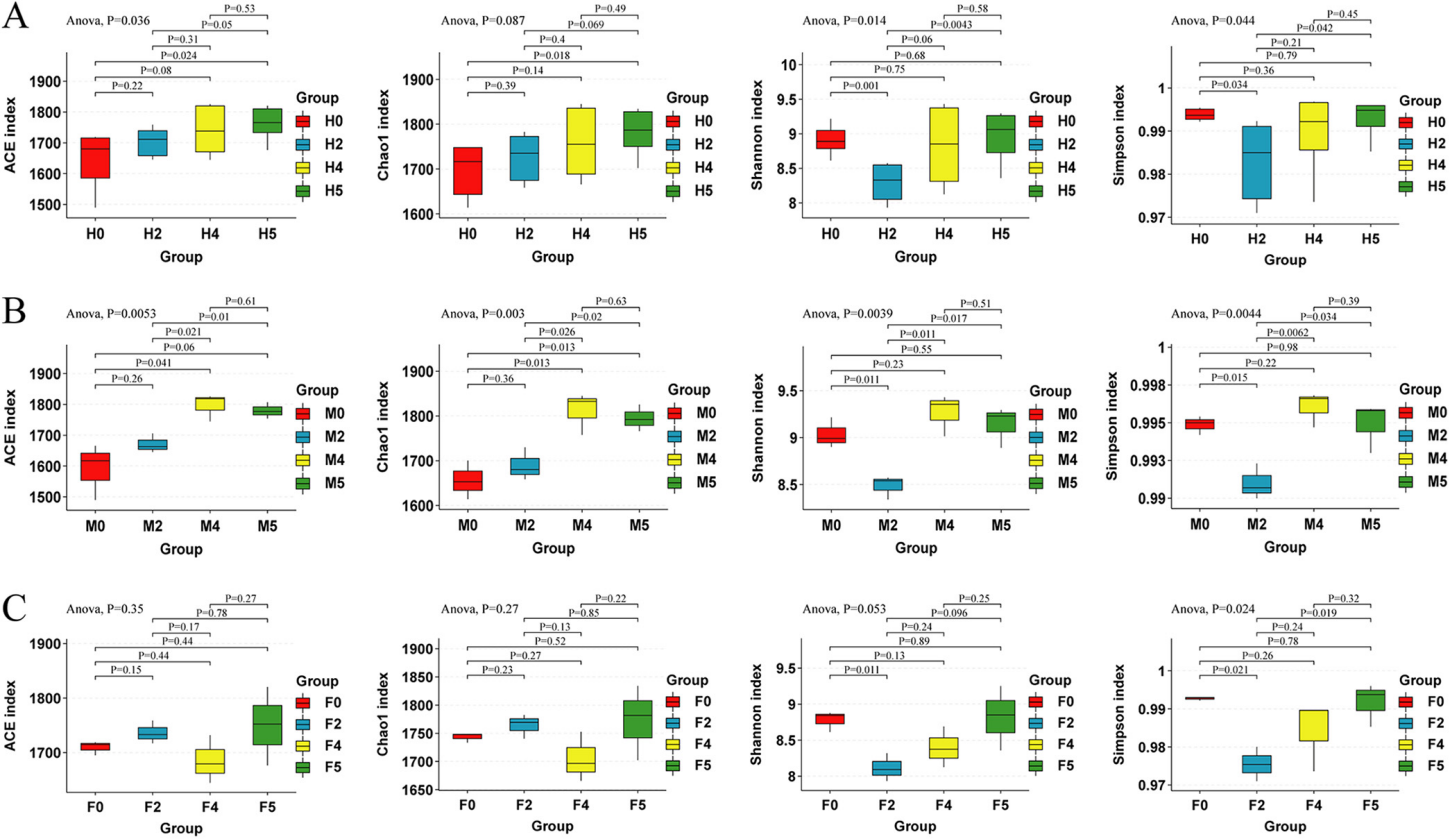
The results of the α-diversity indices. (A) Epiphytic bacteria of *S. thunbergii*. (B) Epiphytic bacteria of male *S. thunbergii*. (C) Epiphytic bacteria of female *S. thunbergii*. An analysis of variance (ANOVA) was used for the statistical comparison of differences between multiple groups, and e a Student’s *t* test was used for thstatistical comparison of differences between two groups (*P < *0.05 indicates a significant difference; *P < *0.01 indicates an extremely significant difference).

Water loss stress had a significant effect on the ACE, Shannon, and Simpson indices in the epiphytic bacterial community of *S. thunbergii* (Fig. 1A) (*P* < 0.05) but had no significant effect on the Chao1 index (Fig. 1A) (*P* > 0.05). With increasing water loss duration, the ACE and Chao1 indices gradually increased, whereas the Shannon index and Simpson index decreased significantly at 2 h of water loss and then increased (Table S2).

10.1128/msphere.00307-22.3 TABLE S2 The α-diversity indices of the epiphytic bacteria of *S. thunbergii.* Download Table S2, DOCX file, 0.01 MB. Copyright © 2022 Sun et al. 2022 Sun et al. https://creativecommons.org/licenses/by/4.0/ This content is distributed under the terms of the Creative Commons Attribution 4.0 International license.

However, the responses of various indicators of epiphytic bacterial communities of male and female *S. thunbergii* to water loss stress were different. In males, with increasing water loss duration, the ACE and Chao1 indices increased significantly and then decreased after rehydration (Table S3; Fig. 1B) (*P* < 0.01), whereas the Shannon index and Simpson index decreased significantly at 2 h of water loss and then increased (Table S3; Fig. 1B) (*P* < 0.01). In females, water loss stress had no significant effect on the ACE, Chao1 or Shannon indices (Fig. 1C) (*P* > 0.05), and only the Simpson index decreased significantly at 2 h of water loss and then increased (Table S3; Fig. 1C) (*P* < 0.05). In addition, the Shannon index and Simpson index in the male algal samples were higher than those in the female algal samples, while the ACE and Chao1 indices in the female samples were higher than those in the male samples (Table S3), indicating that the epiphytic bacteria on female *S. thunbergii* have a higher species richness and that the epiphytic bacteria on male *S. thunbergii* have a higher species evenness.

10.1128/msphere.00307-22.4 TABLE S3 The α-diversity indices of the epiphytic bacteria of male and female *S. thunbergii*. Download Table S3, DOCX file, 0.01 MB. Copyright © 2022 Sun et al. 2022 Sun et al. https://creativecommons.org/licenses/by/4.0/ This content is distributed under the terms of the Creative Commons Attribution 4.0 International license.

### Effect of water loss stress on the β-diversity of the epiphytic bacterial community of *S. thunbergii*.

**FIG 2:**
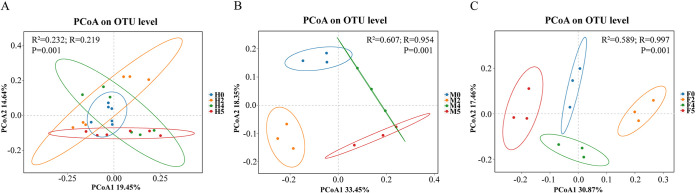
Principal coordinates analysis (PCoA) results based on Bray-Curtis distances. (A) Epiphytic bacteria of *S. thunbergii*. (B) Epiphytic bacteria of male *S. thunbergii*. (C) Epiphytic bacteria of female *S. thunbergii*.

A principal coordinates analysis (PCoA) was used to evaluate the effect of water loss stress on the composition and structure of the microbial community among groups. The calculation results based on Bray-Curtis distances showed that the samples (Fig. 2A) could not be clustered significantly. A permutational analysis of variance (PERMANOVA) showed that there was significant difference in sample grouping and only 23.2% of this difference can be explained by water loss stress (R^2^ = 0.232, *P < *0.05). However, when the experimental groups were analyzed separately according to algal sex, it was found that in experimental groups of a single sex of *S. thunbergii*, the samples within the group were obviously clustered (Fig. 2B and C). A PERMANOVA showed that there were more significant differences in sample grouping (male: R^2^ = 0.607, *P < *0.01; female: R^2^ = 0.589, *P < *0.01), and the results of an analysis of similarities (ANOSIM) showed that the difference between groups was greater than that within groups (male: *R* = 0.954, *P < *0.01; female: *R* = 0.997, *P < *0.01). The above results indicate that water loss stress has significant effect on the β-diversity of the epiphytic bacterial community of *S. thunbergii*, but according to separate sex analyses, water loss stress has more significant effect on the β-diversity of the epiphytic bacterial communities of both male and female *S. thunbergii*.

### Community composition of epiphytic bacteria.

**FIG 3:**
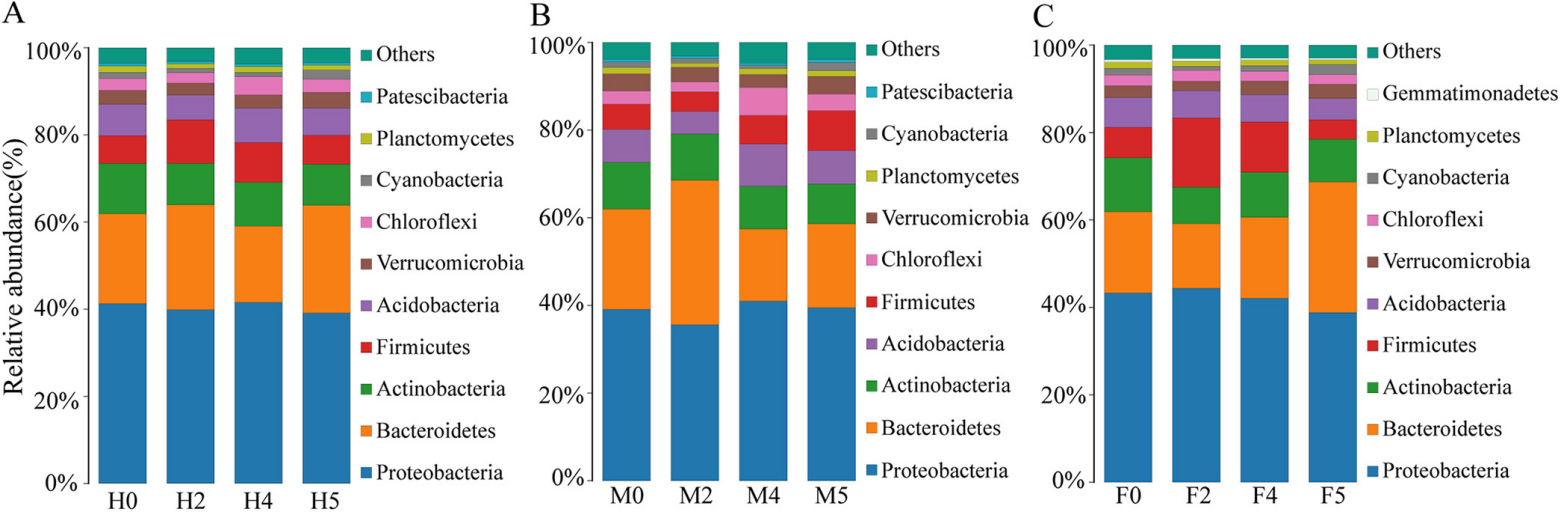
Distribution of the top 10 bacterial taxa with relative abundance at the phylum level. (A) Epiphytic bacteria of *S. thunbergii*. (B) Epiphytic bacteria of male *S. thunbergii*. (C) Epiphytic bacteria of female *S. thunbergii*.

**FIG 4:**
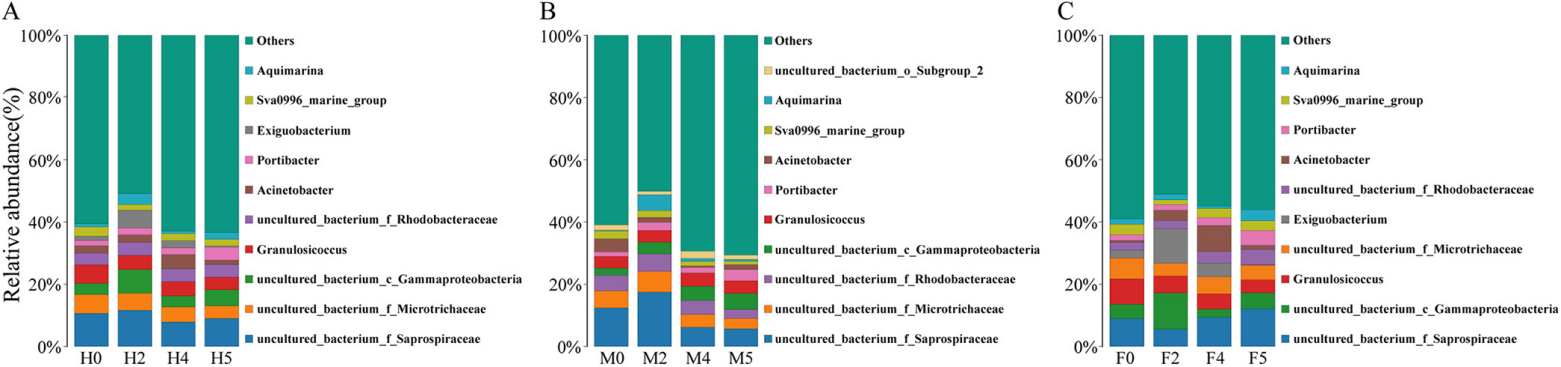
Distribution of the top 10 bacterial taxa with relative abundance at the genus level. (A) Epiphytic bacteria of *S. thunbergii*. (B) Epiphytic bacteria of male *S. thunbergii*. (C) Epiphytic bacteria of female *S. thunbergii*.

A total of 30 phyla, 79 classes, 192 orders, 325 families, 623 genera, and 681 species were identified from all of the samples. Under the condition of water loss stress, the dominant taxa at the phylum level and genus level of each experimental group were basically the same (Fig. 3 and 4). By comparing the abundance of the dominant taxa, it was found that water loss stress had a certain influence on the abundance of the dominant taxa at the phylum level of *S. thunbergii*, but it had a more significant influence at the genus level (*P < *0.05). At the same time, according to separate sex analyses, the dominant taxa of the epiphytic bacteria on male and female algae showed significant differences in response to water loss stress.

### Taxonomic composition at the phylum level.

As shown in Fig. 3, the dominant phyla were almost the same among the groups, including *Proteobacteria*, *Bacteroidetes*, *Actinobacteria*, *Firmicutes*, *Acidobacteria*, and *Verrucomicrobia*, among others. However, the relative abundances of these dominant phyla were different (Table S4). Among them, *Proteobacteria* were the most dominant taxa in each group (35.58% to 44.37%), followed by *Bacteroidetes* (14.78% to 32.95%) and *Actinobacteria* (8.36% to 12.36%). An analysis of differences between groups showed that in the experimental group, without consideration of algal sex, the relative abundance of *Cyanobacteria* only decreased significantly at 2 and 4 h of water loss and then increased again after rehydration (*P < *0.05).

However, in the experimental groups with the consideration of single algal sex, water loss stress had a significant effect on the relative abundance of some dominant bacteria (*P < *0.05). In the male algal samples, the relative abundance of *Cyanobacteria* decreased with increasing water loss duration; the lowest was 0.71% at 4 h and increased to 1.88% after rehydration. The relative abundance of *Firmicutes* decreased significantly at 2 h after water loss, reaching 4.45%, and then increased to 9.03% after rehydration. Interestingly, *Firmicutes* showed the opposite trend in females, with their relative abundance significantly increasing from 6.94% to 15.82% at 2 h of water loss and then decreasing to 4.41% after rehydration. In addition, the relative abundance of *Bacteroidetes* significantly decreased to 14.78% at 2 h of water loss and then increased to 29.87% after rehydration.

### Taxonomic composition at the genus level.

As shown in Fig. 4, the dominant genera among the groups were basically the same (Table S5). These dominant genera were divided into uncultured taxa and culturable taxa. The uncultured taxa include *uncultured_bacterium_f_Saprospiraceae*, *uncultured_bacterium_f_Microtrichaceae*, *uncultured_bacterium_f_Rhodobacteraceae*, and *uncultured_bacterium_c_Gammaproteobacteria*. Culturable taxa include Granulosicoccus, Acinetobacter, Portibacter, Exiguobacterium, Aquimarina, and the *Sva0996 marine group*.

In the experimental group, regardless of the algal sex, *uncultured_bacterium_f_Saprospiraceae* was the most dominant genus in the uncultured taxa (7.86% to 11.65%), while *Granulosicoccus* was the most dominant genus in the culturable taxa (4.09% to 6%). An analysis of differences between the groups showed that water loss stress significantly changed the relative abundances of *Portibacter* and *Aquimarina* (*P < *0.05). With increasing water loss duration, the relative abundances of *Portibacter* increased and continued to increase after rehydration. The abundance of *Aquimarina* increased significantly from 1.01% to 3.47% at 2 h of water loss and then decreased to 0.87%.

In the experimental group of male and female algae separately, *uncultured_bacterium_f_Saprospiraceae* was also the most dominant genus of the uncultured taxa, accounting for 5.67% to 17.46% for the males and 5.56% to 12.12% for the females. Similarly, *Granulosicoccus* was the most dominant genus of the culturable taxa, accounting for 3.72% to 4.3% for the males and 4.13% to 8.1% for the females. Water loss stress had a significant effect on the relative abundance of the dominant genera of epiphytic bacteria on male and female algae, and there were also significant differences between them (*P < *0.05). In the female algal samples, the relative abundance of *Exiguobacterium* increased significantly from 2.43% to 11.12% at 2 h of water loss and then significantly decreased to 0.05%. *Portibacter* did not change significantly before 2 h of water loss and then increased significantly from 1.84% to 4.77% after rehydration. Acinetobacter significantly increased with increasing water loss duration, from 0.56% to 8.34% at 4 h of water loss, and then decreased to 1.31% after rehydration.

However, the relative abundance of *Portibacter* and Acinetobacter also changed significantly in male algal samples, but the trend of change was significantly different from that observed in the female samples. The relative abundance of *Portibacter* increased significantly at 2 h of water loss, decreased at 4 h of water loss, and then increased again after rehydration. Acinetobacter decreased significantly with increasing water loss duration, from 4.20% to 0.49% at 4 h of water loss, and then increased to 1.55% after rehydration. In addition, *Aquimarina* increased significantly from 0.25% to 5.06% at 2 h of water loss and then decreased significantly. The *Sva0996 marine group* decreased with increasing water loss duration and continued to decrease after rehydration. It is worth mentioning that the relative abundance of *Exiguobacterium*, which was higher and significantly changed in the female algal samples, was less than 1% in males.

### Indicator species of epiphytic bacteria of *S. thunbergii*.

**FIG 5:**
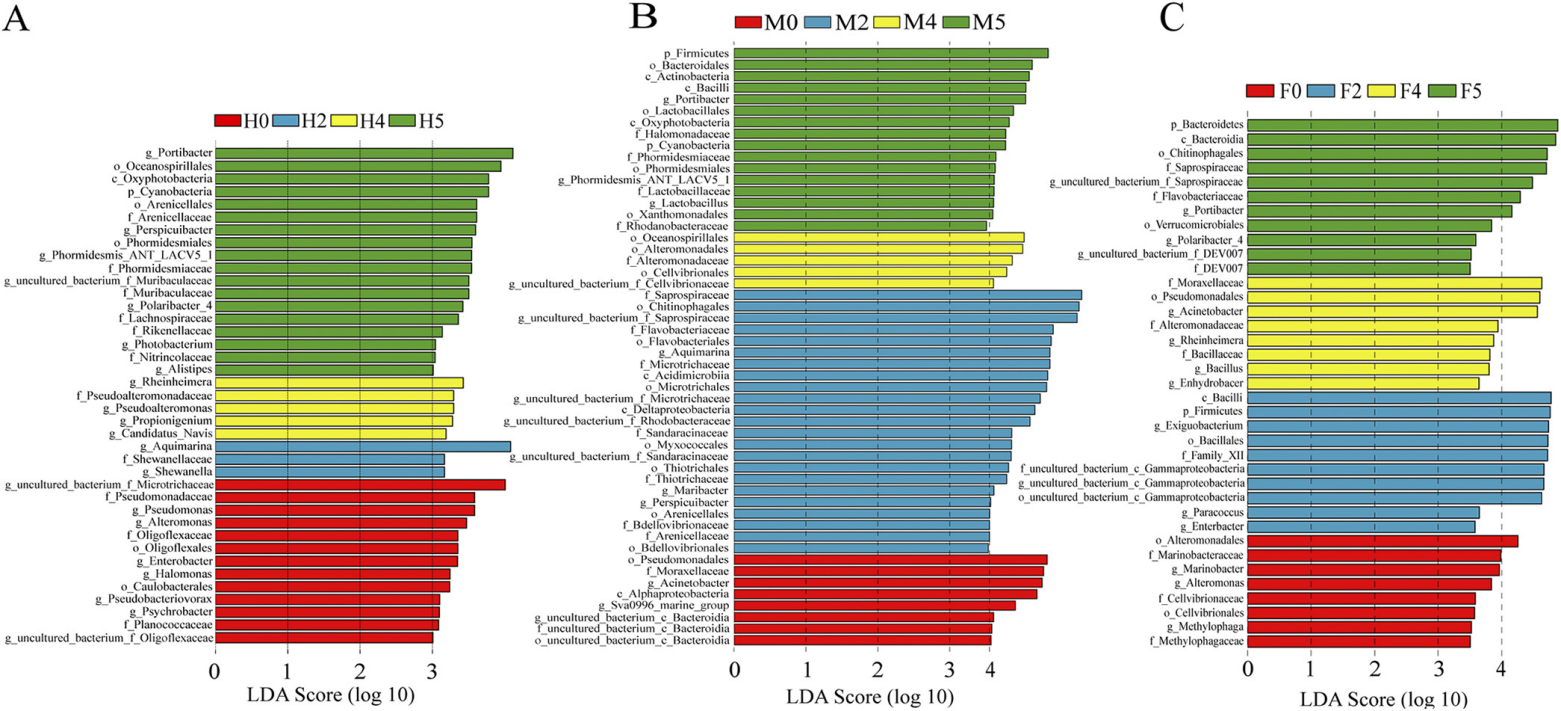
Linear discriminant analysis (LDA) scores of the indicator species in the epiphytic bacterial communities of *S. thunbergii* under different water loss conditions. The colors of the bars represent the groups, and the lengths of the bars represent the contributions of the indicator species (*P < *0.05). (A) Epiphytic bacteria of *S. thunbergii* (LDA > 3.0). (B) Epiphytic bacteria of male *S. thunbergii* (LDA > 3.5). (C) Epiphytic bacteria of female *S. thunbergii* (LDA > 3.5).

In our study, linear discriminant analysis effect size (LEfSe) was used to show the effects of water loss stress on the indicator species of the epiphytic bacteria of *S. thunbergii*. Figure 5A shows that water loss stress significantly reduced the number of indicator species with significant differences in the epiphytic bacteria of *S. thunbergii* (LDA > 3). The representative indicator species of each experimental group is as follows: Pseudomonas and *Alteromonas* at 0 h of water loss, *Aquimarina* and *Shewanella* at 2 h of water loss, *Rheinheimera* and *Pseudoalteromonas* at 4 h of water loss, *Portibacter* and *Oceanospirillales* (order) at 1 h of rehydration.

However, the effects of water loss stress on the indicator species of epiphytic bacteria of male and female *S. thunbergii* were different from the above results, and there were significant differences between males and females (LDA > 3.5). Figure 5B shows that the number of indicator species of male algae epiphytic bacteria increased significantly at 2 h of water loss and decreased significantly at 4 h of water loss, but there was no significant change in females at each stage (Fig. 5C). In addition, the number of indicator species with significant differences in males was significantly higher than that observed in females, and the indicator species of each stage in the males were different from those of the females. The representative indicator species of each experimental group in males is as follows: Acinetobacter and *Sva0996 marine group* at 0 h of water loss, *Saprospiraceae* (family) and *Aquimarina* at 2 h of water loss, *Oceanospirillales* (order) and *Alteromonadaceae* (family) at 4 h of water loss, *Firmicutes* (phylum) and *Portibacter* at 1 h of rehydration. The representative indicator species of each experimental group in females is as follows: *Alteromonas* and *Marinobacter* at 0 h of water loss, *Firmicutes* (phylum) and *Exiguobacterium* at 2 h of water loss, Bacillus and Acinetobacter at 4 h of water loss, *Portibacter* and *Flavobacteriaceae* (family) at 1 h of rehydration.

### Functional prediction of epiphytic bacteria in *S. thunbergii*.

**FIG 6:**
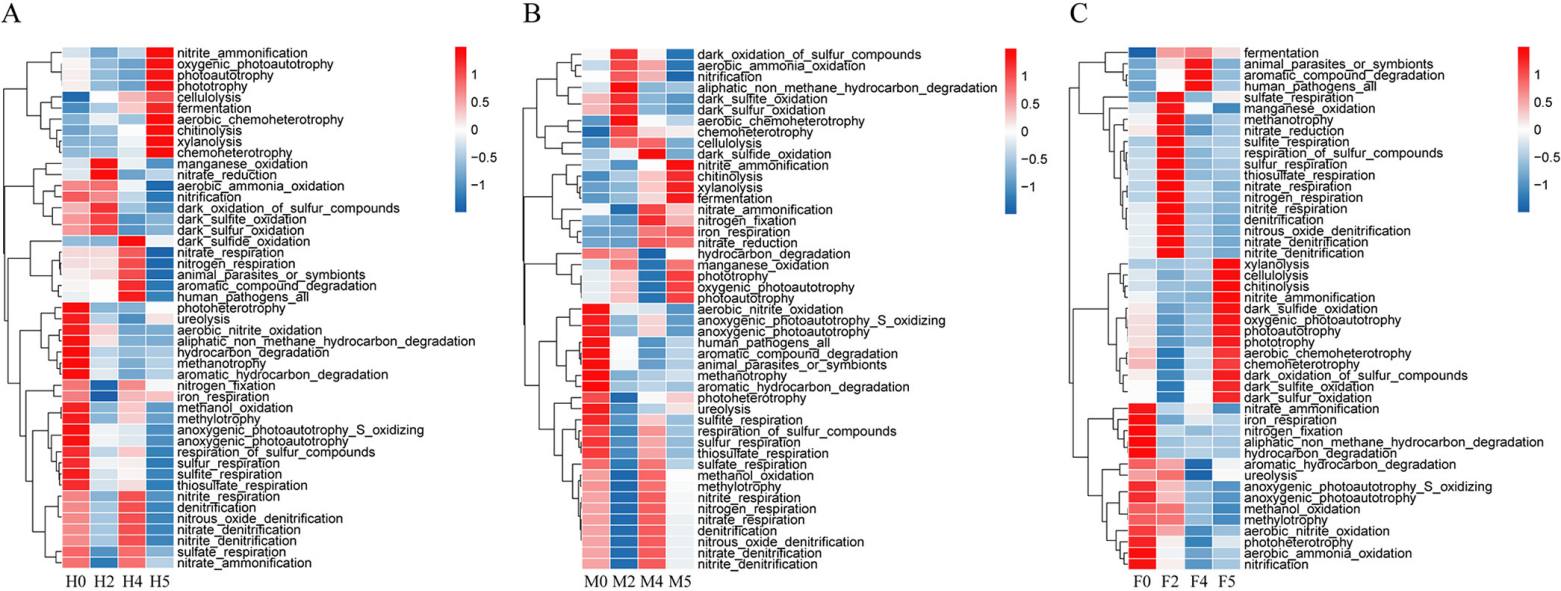
The heatmap of functional abundances predicted by FAPROTAX, based on the SILVA database. The vertical axis indicates the sample groups at different water loss times. The horizontal axis indicates each functional group of the elemental cycle. Red and blue indicate the functional abundance; the larger the value, the higher the predicted functional abundance. (A) Epiphytic bacteria of *S. thunbergii.* (B) Epiphytic bacteria of male *S. thunbergii.* (C) Epiphytic bacteria of female *S. thunbergii*.

The Functional Annotation of Prokaryotic Taxa (FAPROTAX) database was used to analyze the 16S rRNA data of the epiphytic bacteria on *S. thunbergii* to predict the functions of bacterial communities under different water loss conditions. 57 functional types were detected in the samples, of which 19 were associated with carbon metabolism, 15 with nitrogen metabolism, and 9 with sulfur metabolism. These metabolism-related functional types were processed to generate a functional abundance heatmap (Fig. 6), which clearly visualizes the effects of water loss stress on metabolism-related functions. In the experimental group, regardless of sex, samples with functions in cellulolysis, xylanolysis, chitinolysis, aromatic compound degradation, fermentation, chemoheterotrophy, nitrate respiration, nitrogen respiration, and dark sulfide oxidation increased in abundance with increasing water loss duration. Samples with functions in nitrogen fixation, nitrate ammonification, nitrate respiration, sulfate respiration, and the denitrification of nitrogen compounds decreased at 2 h of water loss and increased at 4 h of water loss. In addition, the abundance of other predicted functions related to carbon, nitrogen, and sulfur metabolism decreased by varying degrees.

Interestingly, when analyzed separately by sex, it was found that the predicted functional abundances of the epiphytic bacteria on male and female algae changed obviously, and there were obvious differences. For carbon metabolism, the functional abundance increased with increasing water loss duration for chitinolysis, xylanolysis, fermentation, cellulolysis, and chemoheterotrophy in the male algal samples and for fermentation, methanotrophy, and aromatic compound degradation in the female algal samples. For nitrogen metabolism, the functional abundances of nitrite ammonification, nitrogen fixation, nitrate reduction, aerobic ammonia oxidation, and nitrification increased with increasing water loss duration in the male algal samples. In addition, nitrate ammonification and functional abundances related to respiration and the denitrification of nitrogen compounds decreased at 2 h of water loss and increased at 4 h of water loss in the male algal samples. However, the trends of these functional abundances of epiphytic bacteria on female algal samples were the opposite. Similar results also occurred for sulfur metabolism. The functional abundances of samples related to the respiration of sulfur and sulfur compounds by epiphytic bacteria decreased at 2 h of water loss and increased at 4 h of water loss in the male algal samples, while these functional abundances increased at 2 h of water loss and decreased at 4 h of water loss in the female algal samples. Other metabolic functions related to carbon, nitrogen, and sulfur metabolism of epiphytic bacteria on the male and female samples decreased to varying degrees. However, the functional abundances associated with animal and human diseases (such as human pathogens all and animal parasites or symbols) increased in females and decreased in males.

## DISCUSSION

The epiphytic bacterial communities on the surfaces of macroalgae have high host specificities (26), but at the same time, the community compositions and diversities are affected by environmental factors (3, 20, 29). There have been many reports on the influence of water reduction on epiphytic bacterial communities on higher plants. For example, Bechtold et al. (30) found that the microbial community diversity and composition of the abundance of dominant bacteria on forage grasses were significantly altered under drought conditions. Similar results were reported both in *Populus* trees (31) and in the peanut rhizosphere (32) under drought stress. In this study, high-throughput sequencing technology was used to explore the effects of water loss stress caused by tidal effects on the structure and function of the epiphytic bacterial community of *S. thunbergii* in the intertidal zone and to compare the different responses of the epiphytic bacterial communities of male and female algae to water loss stress.

### Effects of water loss stress on the epiphytic bacterial community of *S. thunbergii*.

In this study, water loss stress had a significant effect on the α-diversity indices, which increased the abundance of epiphytic bacteria after water loss. This is consistent with the result that long-term drought increased the bacterial richness in the foliar phyllosphere of Quercus ilex (33). It may be that the tide caused water loss and involved a change in oxygen conditions such that the abundance of the inhibited epiphytic bacteria of *S. thunbergii* increased during water loss exposure, and there may also be some new bacteria from the air environment that adhere to the surfaces of the algae. However, the diversity of bacteria first decreased and then increased, which suggested that some drought-intolerant marine bacteria decreased during water loss. For example, both *Cyanobacteria* and Gemmatimonadetes have been reported to be sensitive to drought, and their abundances decreased after water loss in previous studies (34, 35). But, with the increase in water loss duration, the number of some drought-resistant bacteria increased, which led to an increase in diversity. So far, *Actinobacteria* and Chloroflexi have been reported to be dominant in drought environments (35, 36), whereas *Proteobacteria* and *Firmicutes* were found to be significantly enriched after drought treatment (34).

In addition, water loss stress also had an effect on the abundance of dominant taxa of epiphytic bacteria. The abundance of *Cyanobacteria* decreased with increasing water loss duration, which was consistent with the conclusion of Liu et al. (34) that drought stress changed the abundance of microorganisms in bulk soil and in plant root sheaths. It has been reported that the decrease in *Cyanobacteria* abundance may be caused by its sensitivity to drought stress (37). At the genus level, the relative abundance of *Portibacter* showed an overall increasing trend with increasing water loss duration. A large number of previous reports showed that *Portibacter* was mostly found in the marine environment and did not exist in a free state, and it was later confirmed that it was more inclined to live in a fixed state (38, 39). In addition, it was reported that *Aquimarina* was significantly enriched during the degradation processes of Laminaria japonica (40) and Colpomenia sinuosa (41), while in this study, the abundance of *Aquimarina* changed significantly at 2 h of water loss, which may have been due to the change in algal morphology caused by water loss stress (7), resulting in an increase in its abundance. However, the bacteria themselves may not be resistant to drought, resulting in a decrease in abundance in the subsequent time of water loss.

FAPROTAX showed that there were 43 metabolic functions related to carbon, nitrogen, and sulfur, indicating that the epiphytic bacteria of *S. thunbergii* widely participate in the biogeochemical cycle of carbon, nitrogen, and sulfur on the surface of algae and in the environment. McIlroy et al. (38) reported that the *Saprospiraceae* taxa of *Portibacter* can hydrolyze and utilize complex carbon sources. Ooi et al. (42) and Sun et al. (43) reported that *Aquimarina* can degrade chitin and decompose cellulose. LEfSe showed that Pseudomonas, *Aquimarina*, Shewanella, *Oceanospirillales* (order) and *Portibacter* were the indicator species with significant differences in each stage, and these taxa were also reported to be extensively involved in the above-mentioned carbon (44), nitrogen (45, 46), and sulfur metabolism (47). In addition, the predicted functional genes in the epiphytic bacteria on *S. thunbergii* involved in chemoheterotrophy, xylanolysis, cellulolysis, oxidation-reduction, and sulfide and nitrate oxidation were enriched in the process of water loss. It has been reported that the intertidal green alga Ulva pertusa can regulate its cellular osmolarity and maintain cellular water to resist water loss stress by increasing the concentrations of osmoregulators, such as soluble sugars and proline (48). Similarly, an intertidal red alga Gracilaria corticate resisted drought-induced oxidative damage through higher contents of insoluble putrescine and spermine together with enhanced polyunsaturated fatty acids (PUFAs) and fatty acids (49). However, the linkage of metabolic changes between algal epiphytic bacteria and their hosts needs to be further explored.

### Effect of water loss stress on epiphytic bacterial communities in male and female *S. thunbergii*.

When studying the effects of water loss stress on the epiphytic bacterial community of *S. thunbergii* by sex, we were surprised to find that the responses of male and female algal epiphytic bacteria to water loss stress were significant, and some changes were not observed in the experimental group regardless of sex. More importantly, there were differential responses between the samples from male and female *S. thunbergii*.

Water loss stress also caused the diversity of epiphytic bacteria on male and female *S. thunbergii* to decrease at first and then increase. However, the difference was that the change in the Ace, Chao1, and Shannon indices in the males was greater than that observed in the females (Table S3). It was speculated that the male algal epiphytic bacteria were more sensitive to water loss stress. However, previous reports have shown that under drought conditions, the females of most plants are more sensitive than male plants and suffer more negative effects on growth characteristics, photosynthetic efficiency, and reactive oxygen systems (22, 50). Therefore, this study speculated that when faced with external stress, the algae and epiphytic bacteria interacted to resist the adverse environment together, and the epiphytic bacteria on female algae with poor defenses had higher stress resistances, which may be a compensatory mechanism that has evolved between the bacteria and the algae.

The abundance of *Cyanobacteria* in male *S. thunbergii* changed notably, so although it did not change significantly in the female algal samples, the abundance of *Cyanobacteria* changed significantly in the experimental groups regardless of algal sex. In addition, the increased abundance of *Firmicutes* may have occurred because some bacteria help plants improve drought resistance by not only by stimulating physiological responses and regulating abscisic acid levels (51) but also by forming thicker peptidoglycan cell walls to cope with the effects of drought (52). However, we observed that *Firmicutes* had opposite trends in samples from male and female *S. thunbergii*, and the differences in the trends of these bacterial groups after water loss in male and female algae were unclear and need to be studied further.

At the genus level, the common taxa with significant changes in bacteria from both male and female *S. thunbergii* mainly included *Portibacter* and Acinetobacter. *Portibacter* showed an increasing trend in both male and female algal samples; however, Acinetobacter decreased with increasing water loss duration in male algal samples but increased in female algal samples. We speculated that male plants have a stronger defense ability under water loss stress (53, 54) and that some extracellular secretions produced in response to water loss stress are not conducive to the attachment of Acinetobacter. Under the low defense ability of females, Acinetobacter can improve its drought resistance by overexpressing membrane proteins and periplasmic proteins (55, 56) to increase its abundance. It is precisely because of the opposite change in Acinetobacter abundance in male and female algae that no significant change in the bacteria was observed in the experimental group regardless of sex.

In addition, the male and female algae also had their own unique dominant taxa with significant changes, such as *Aquimarina* and the *Sva0996 marine group* in males as well as *Exiguobacterium* in females. The abundance of *Aquimarina* in males increased significantly at 2 h of water loss, which was consistent with the change in trend of *Aquimarina* in the experimental group regardless of sex. However, there are relatively few reports about the *Sva0996 marine group* at present. Papadatou et al. (39) found that the *Sva0996 marine group* was enriched on the surface of a marine anti-pollution control coating and was also abundant during the outbreak of phytoplankton blooms (57) and during the degradation of some algae (40, 41). *Exiguobacterium* had the characteristics of high abundance and significant variation in the female samples but not in the male samples. It is speculated that this may have been related to the specific selection of male and female algae. Many previous studies, such as those of López et al. (58), Etemadifar et al. (59), and Liu et al. (60), have reported that *Exiguobacterium* has the characteristics of drought tolerance, UV radiation resistance, and salt tolerance. This also explains the increased abundance of *Exiguobacterium* on female *S. thunbergii*.

From the comparison of the functional abundance of samples from male and female algae, we speculated that the epiphytic bacteria of male *S. thunbergii* participated in the biogeochemical cycle of carbon, nitrogen, and sulfur. *Geodermatophilus* (*Actinomycetes*) (61), which is related to manganese oxidation, was significantly higher in the female samples than in the male samples, which also led to a higher abundance of manganese oxidation functions in females than in males. Moreover, the functional abundance of pathogenicity was more significant in the female samples. It is speculated that female algae are more likely to be colonized by pathogenic bacteria while suffering from environmental stress. In addition, under the condition of water loss stress, the same functions in male and female samples showed different or even opposite changes in trends, which further shows that species with sex differences will produce different adaptive strategies when faced with environmental stress (50, 54). This is also the reason for the different or insignificant changes in functional abundance in the experimental group regardless of sex.

Thus, in this study, when the effects of water loss stress on the epiphytic bacterial community of *S. thunbergii* were studied regardless of sex, some significant changes between males and females were covered up or offset, resulting in an inability to accurately reflect the effect of water loss stress on the epiphytic bacterial community of *S. thunbergii*. Therefore, for dioecious plants, male and female individuals should be studied separately for better analysis of the responses of plants to environmental stresses.

### Conclusion.

In this study, the changes in the community structure and function of the epiphytic bacteria of *S. thunbergii* under the condition of water loss and the differences between male and female algal samples were discussed. We confirmed that water loss stress had a significant effect on the epiphytic bacterial community of S. *thunbergii* and that this effect had obvious sex-based differences. In the process of water loss under tidal effects, the diversity of the epiphytic bacterial community first decreased and then increased, and the abundance of the dominant taxa, the indicator species, and metabolic functions changed. More importantly, the changes in diversity, the abundance of dominant taxa, the indicator species and the metabolic functions of the male algal epiphytic bacterial community were more significant than those of the female community, indicating that the male algal epiphytic bacteria were more sensitive to the water loss stress. This may have been because the epiphytic bacteria on the male algae are more likely to adapt to environmental stress directly through changes in the taxa themselves or indirectly through changes in the microenvironment between the bacteria and the algae. These results highlight the response characteristics of the intertidal macroalgae epiphytic bacterial community after water loss stress and reveal that the response of the epiphytic bacterial community to stress is related to the sex of the host plants.

## MATERIALS AND METHODS

### Sample collection and processing.

**FIG 7:**
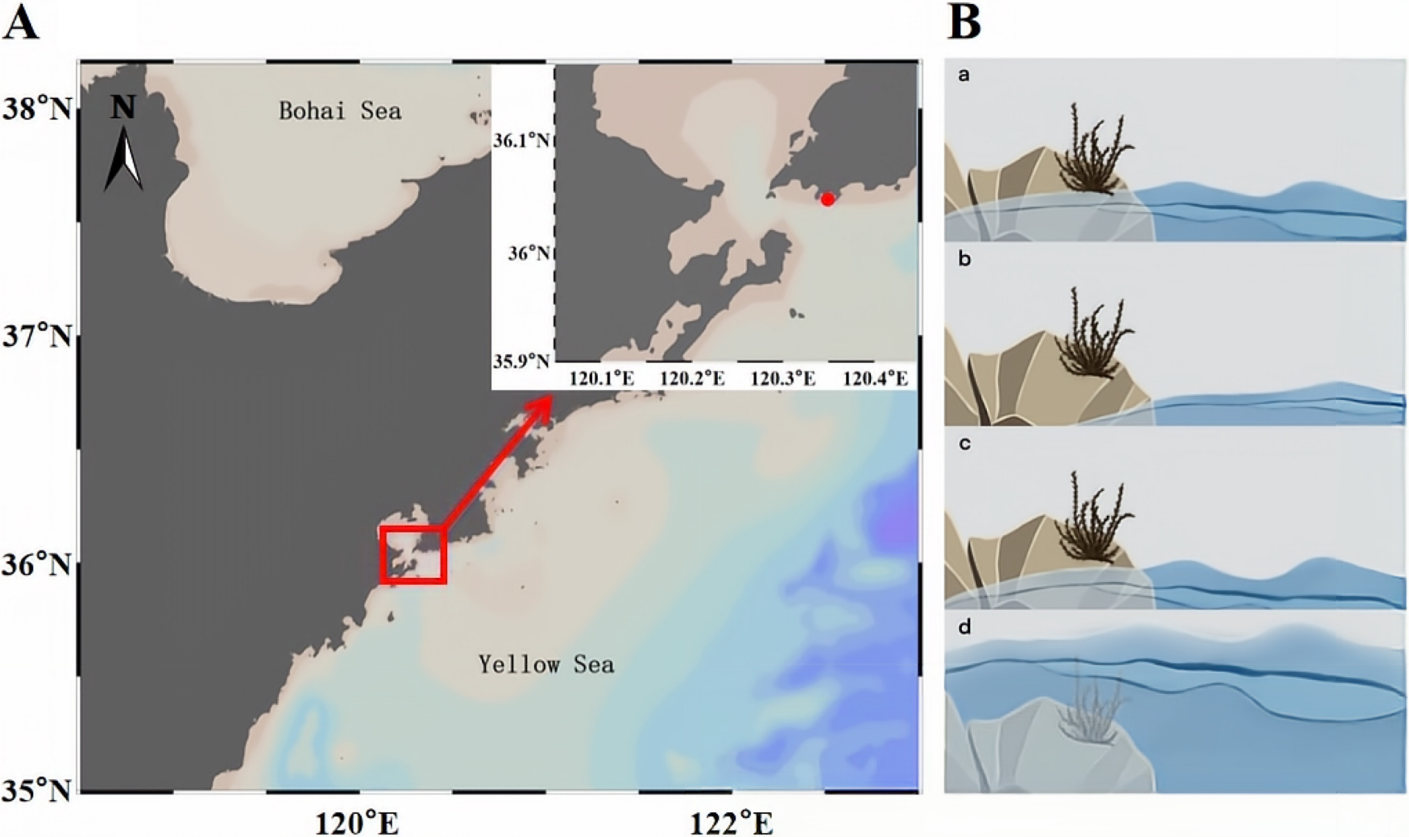
Sample information. (A) Sampling location (red dot indicates the sampling area). (B) The state of *S. thunbergii* at different stages of water loss (a: water loss for 0 h; b: water loss for 2 h; c: water loss for 4 h; d: rehydration for 1 h).

The sampling site is located in the intertidal zone along the coast of Taiping Bay in Qingdao (Fig. 7), 120°35’ E, 36°05’ N. The area experiences a regular, semidiurnal tide, with two high tides and two low tides every day, and the mean height of the tide is 2.8 m. On the evening of July 15th, 2021, 24 strains of *S. thunbergii* (12 males and 12 females) with basically the same growth were randomly marked in a rectangular area of approximately 30 m × 5 m in the intertidal zone, and these samples were randomly divided into four groups, each with 6 strains of *S. thunbergii* (3 males and 3 females). The male and female *S. thunbergii* were identified according to the morphological characteristics of the receptacles first and then were determined via microscopic examination, according to the difference of the receptacle structure. Those individuals weighing about 25 g were selected for further treatment. Each group of *S. thunbergii* was collected at 0, 2, and 4 h after water loss at low tide and 1 h after rising tide (Fig. 7B), put into sterilized sample bags, and preserved in a portable box with ice bags.

The samples of *S. thunbergii* were simply washed with sterile seawater. Then, 25 g of the samples were weighed and put into sterilized 250 mL conical bottles, and 70 mL phosphate-buffered saline (PBS, 0.01 mmol/L) were added. Then, the bottles were sealed with sterile membranes and shaken (200 rpm) for 30 min at room temperature. After shaking, the obtained suspension was filtered with a sterile 500-mesh sieve to remove the mixed sediment and other impurities. Then, the eluent was filtered in a sterile environment with a vacuum suction filter, and the epiphytic bacteria were collected onto a 0.22 μm filter membrane. Then, the membrane was put into a sample tube and stored at −80°C (62). Finally, the samples were placed on dry ice and sent to BioMarker Technologies (Beijing, China) for sequencing. *S. thunbergii* samples from four stages were marked as H0, H2, H4, and H5, with 6 parallel samples in each group (3 males and 3 females). The male samples of *S. thunbergii* in each stage were marked as M0, M2, M4, and M5, and the female samples in each stage were marked as F0, F2, F4, and F5.

### DNA extraction, PCR amplification and high-throughput sequencing.

High-throughput sequencing was conducted by BioMarker Technologies. The total DNA of epiphytic bacteria in each sample was isolated according to the instructions of TGuide S96 Magnetic Soil And Stool DNA Kit (TIANGEN BIOTECH, BEIJING), and then the purity and concentration of the total DNA of the epiphytic bacteria of *S. thunbergii* were detected using a NanoDrop 2000 UV spectrophotometer (Thermo Fisher Scientific, USA). After the detected DNA was removed, the V3 + V4 region of the 16S rRNA genes was amplified with 338F primer (5′-ACTCCTACGGGAGGCAGCA-3′) and 806R primer (5′-GGACTACHVGGGTWTCTAAT-3′). Polymerase chain reaction (PCR) amplification was performed under the following conditions: 95°C for 5 min, 95°C for 30 s, 50°C for 30 s, and 72°C for 40 s for 25 cycles and then 72°C for 7 min and storage at 4°C. Then, the PCR products were recovered using agarose gel electrophoresis (1.8%) and detected by purification, elution, and agarose electrophoresis (1.8%) (120 V, 40 min). The 16S rRNA of bacteria was high-throughput sequenced using the Illumina NovaSeq 6000 sequencing platform.

### Statistical analysis.

The raw data were spliced using FLASH (version 1.2.11) (63). The spliced sequences were quality filtered using Trimmomatic (version 0.33) (64). Chimeras were removed using UCHIME (version 8.1) (65), and then the sequences corresponding to “chloroplast” and “mitochondria” were removed (66) to obtain high-quality, valid data. Sequences were clustered at a 97% similarity level using USEARCH (version 10.0) (67), and OTUs were filtered using a threshold of 0.005% of all sequences. The obtained OTUs were taxonomically matched, and species were annotated using the Silva (Release128) database with an annotation confidence threshold of 0.8 (68, 69). The Ace, Chao1, Shannon, and Simpson indices at the OTU level of the samples were calculated using Mothur (version 1.30) software (70), and boxplots were drawn to illustrate the significance of the α-diversity. Both Student’s *t* tests and analyses of variance (ANOVA) were used to test the significance of the differences between groups. The QIIME software, based on the Bray-Curtis method, was used for β-diversity analysis. A principal coordinates analysis (PCoA) was used to evaluate sample changes in the microbial community structure (71, 72), permutational multivariate analyses of variance (PERMANOVA) were used to evaluate the degree of grouping among sample differences, and an analysis of similarities (ANOSIM) was used to compare the intragroup and intergroup differences of samples (11). LEfSe was used to analyze the OTU data to reveal taxa that significantly contributed to the differences observed between the samples. The statistical tests and analyses of variance were performed by combining the nonparametric factorial Kruskal-Wallis (KW) rank-sum test, the Wilcoxon rank-sum test, and linear discriminant analysis (LDA) simultaneously at all taxonomic levels to identify the stable different taxa between the different groups, which are the “indicator species”. The LDA scores were used to estimate the differently abundant taxa between groups. An analysis of the indicator species was performed in R (package “ggplot2”) and was conducted from the genus to phylum levels (73, 74). The sequencing data of the epiphytic bacteria on *S. thunbergii* under different water loss conditions were functionally annotated using the FAPROTAX database, which is a tool that maps prokaryotic clades to establish metabolic or other ecologically relevant functions based on the current literature on cultured strains (75, 76).

### Data availability.

The bacterial sequences obtained in this study have been saved to the National Center for Biotechnology Information (NCBI) with BioProject ID: PRJNA836712.

10.1128/msphere.00307-22.5 TABLE S4 The top 10 relative abundances of epiphytic bacteria of *S. thunbergii* in different groups at the phylum level (%). Download Table S4, DOCX file, 0.01 MB. Copyright © 2022 Sun et al. 2022 Sun et al. https://creativecommons.org/licenses/by/4.0/ This content is distributed under the terms of the Creative Commons Attribution 4.0 International license.

10.1128/msphere.00307-22.6 TABLE S5 The top 10 relative abundances of epiphytic bacteria of *S. thunbergii* in different groups at the genus level (%). Download Table S5, DOCX file, 0.01 MB. Copyright © 2022 Sun et al. 2022 Sun et al. https://creativecommons.org/licenses/by/4.0/ This content is distributed under the terms of the Creative Commons Attribution 4.0 International license.
