# Supplementary figures and images for: Effects of Water Loss Stress under Tidal Effects on the Epiphytic Bacterial Community of Sargassum thunbergii in the Intertidal Zone
Source: mSphere. 2022 Sep 29;7(5):e00307-22. doi: 10.1128/msphere.00307-22 (PMC9599519; doi:10.1128/msphere.00307-22)

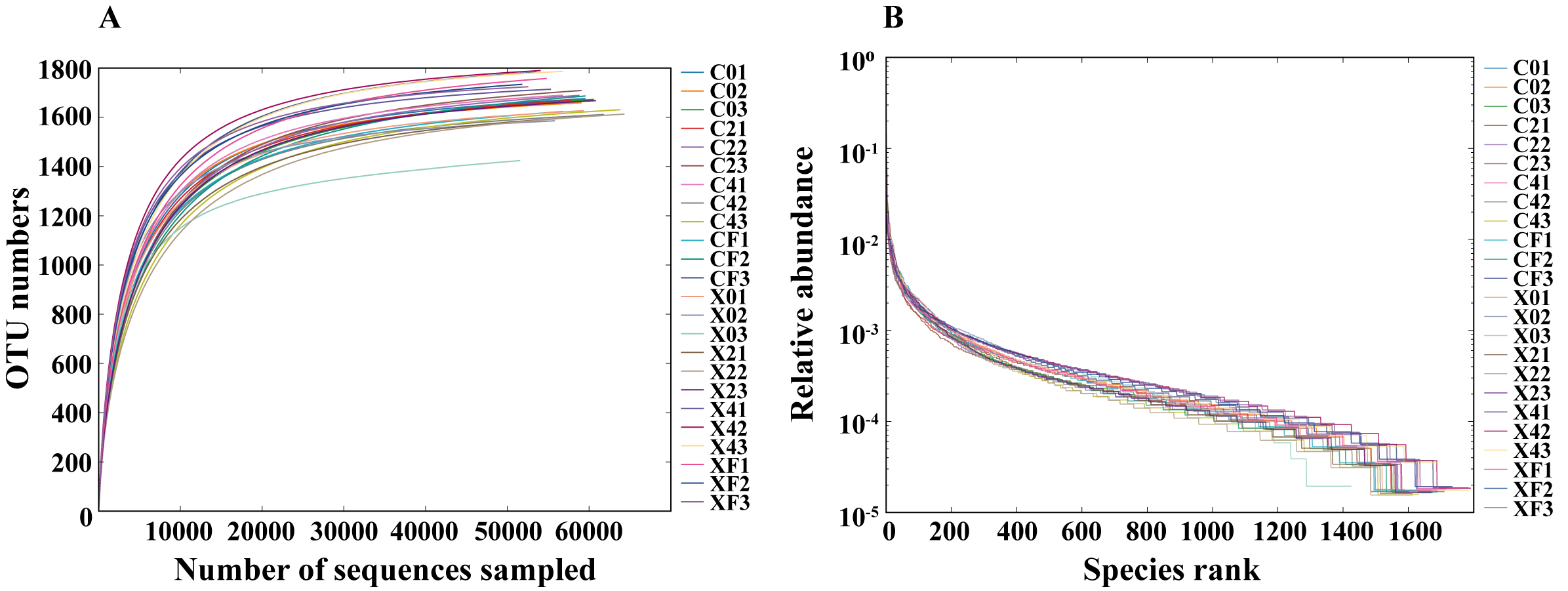

Supplement: FIG S1 [file msphere.00307-22-s0001.tif]
