# Supplementary material for: Effects of Water Loss Stress under Tidal Effects on the Epiphytic Bacterial Community of Sargassum thunbergii in the Intertidal Zone
Source: mSphere. 2022 Sep 29;7(5):e00307-22. doi: 10.1128/msphere.00307-22 (PMC9599519; doi:10.1128/msphere.00307-22)
Supplement: TABLE S1 [file msphere.00307-22-s0002.docx]

| Sample ID | Raw Reads | Clean Reads | Effective Reads |
| --- | --- | --- | --- |
| C01 | 80147 | 79854 | 77559 |
| C02 | 80130 | 79830 | 77658 |
| C03 | 79904 | 79598 | 77283 |
| X01 | 80105 | 79836 | 77462 |
| X02 | 80510 | 80236 | 77228 |
| X03 | 80174 | 79883 | 75806 |
| C21 | 80020 | 79766 | 76950 |
| C22 | 79935 | 79650 | 77804 |
| C23 | 79840 | 79575 | 77289 |
| X21 | 79761 | 79499 | 76265 |
| X22 | 79867 | 79594 | 77098 |
| X23 | 79986 | 79731 | 77286 |
| C41 | 80043 | 79799 | 77225 |
| C42 | 79957 | 79680 | 77851 |
| C43 | 80180 | 79897 | 77718 |
| X41 | 80008 | 79719 | 76561 |
| X42 | 79875 | 79617 | 76720 |
| X43 | 80232 | 79971 | 77311 |
| CF1 | 79909 | 79609 | 76758 |
| CF2 | 79955 | 79659 | 77259 |
| CF3 | 79922 | 79637 | 76677 |
| XF1 | 79861 | 79571 | 75968 |
| XF2 | 80265 | 79990 | 76572 |
| XF3 | 80089 | 79820 | 76908 |
