# Supplementary material for: Effects of Water Loss Stress under Tidal Effects on the Epiphytic Bacterial Community of Sargassum thunbergii in the Intertidal Zone
Source: mSphere. 2022 Sep 29;7(5):e00307-22. doi: 10.1128/msphere.00307-22 (PMC9599519; doi:10.1128/msphere.00307-22)
Supplement: TABLE S2 [file msphere.00307-22-s0003.docx]

| Group | Ace | Chao1 | Simpson | Shannon |
| --- | --- | --- | --- | --- |
| H0 | 1650.2887 | 1699.5157 | 0.9938 | 8.9069 |
| H2 | 1703.8306 | 1726.8259 | 0.9832 | 8.2993 |
| H4 | 1740.7753 | 1758.3793 | 0.9902 | 8.8312 |
| H5 | 1764.6823 | 1783.6625 | 0.9933 | 8.9793 |
