# Supplementary material for: Effects of Water Loss Stress under Tidal Effects on the Epiphytic Bacterial Community of Sargassum thunbergii in the Intertidal Zone
Source: mSphere. 2022 Sep 29;7(5):e00307-22. doi: 10.1128/msphere.00307-22 (PMC9599519; doi:10.1128/msphere.00307-22)
Supplement: TABLE S3 [file msphere.00307-22-s0004.docx]

| Group | Ace | Chao1 | Simpson | Shannon |
| --- | --- | --- | --- | --- |
| F0 | 1709.5731 | 1743.2248 | 0.9927 | 8.7776 |
| F2 | 1736.3192 | 1764.1223 | 0.9755 | 8.1138 |
| F4 | 1685.2702 | 1704.9481 | 0.9843 | 8.3962 |
| F5 | 1749.7701 | 1772.7398 | 0.9917 | 8.8197 |
| Total female | 1720.2332 | 1746.2588 | 0.9861 | 8.5268 |
| M0 | 1591.0043 | 1655.8065 | 0.9949 | 9.0363 |
| M2 | 1671.3421 | 1689.5295 | 0.9910 | 8.4847 |
| M4 | 1796.2804 | 1811.8106 | 0.9960 | 9.2663 |
| M5 | 1779.5945 | 1794.5852 | 0.9949 | 9.1389 |
| Total male | 1709.5553 | 1737.9330 | 0.9942 | 8.9816 |
