# Supplementary material for: Effects of Water Loss Stress under Tidal Effects on the Epiphytic Bacterial Community of Sargassum thunbergii in the Intertidal Zone
Source: mSphere. 2022 Sep 29;7(5):e00307-22. doi: 10.1128/msphere.00307-22 (PMC9599519; doi:10.1128/msphere.00307-22)
Supplement: TABLE S4 [file msphere.00307-22-s0005.docx]

| Phylum | H0 | H2 | H4 | H5 |
| --- | --- | --- | --- | --- |
| *Proteobacteria* | 41.26 | 39.88 | 41.53 | 39.12 |
| *Bacteroidetes* | 20.66 | 24.08 | 17.55 | 24.71 |
| *Actinobacteria* | 11.55 | 9.50 | 10.02 | 9.46 |
| *Firmicutes* | 6.36 | 10.00 | 9.14 | 6.64 |
| *Acidobacteria* | 7.15 | 5.67 | 7.88 | 6.20 |
| *Verrucomicrobia* | 3.25 | 2.75 | 3.03 | 3.65 |
| *Chloroflexi* | 2.76 | 2.41 | 4.21 | 3.00 |
| *Cyanobacteria* | 1.39 | 0.99 | 1.03 | 2.11 |
| *Planctomycetes* | 1.41 | 1.05 | 1.29 | 1.14 |
| *Patescibacteria* | 0.63 | 0.51 | 0.56 | 0.60 |
| Phylum | M0 | M2 | M4 | M5 |
| *Proteobacteria* | 39.09 | 35.59 | 40.96 | 39.46 |
| *Bacteroidetes* | 22.88 | 32.95 | 16.47 | 19.19 |
| *Actinobacteria* | 10.68 | 10.60 | 9.47 | 9.08 |
| *Acidobacteria* | 7.51 | 5.13 | 9.69 | 7.62 |
| *Firmicutes* | 5.74 | 4.45 | 6.47 | 9.03 |
| *Chloroflexi* | 3.07 | 2.33 | 6.39 | 3.84 |
| *Verrucomicrobia* | 3.87 | 3.31 | 2.96 | 4.06 |
| *Planctomycetes* | 1.45 | 0.91 | 1.37 | 1.28 |
| *Cyanobacteria* | 1.22 | 1.07 | 0.71 | 1.88 |
| *Patescibacteria* | 0.68 | 0.62 | 0.55 | 0.69 |
| Phylum | F0 | F2 | F4 | F5 |
| *Proteobacteria* | 43.29 | 44.37 | 42.06 | 38.81 |
| *Bacteroidetes* | 18.60 | 14.78 | 18.53 | 29.87 |
| *Actinobacteria* | 12.36 | 8.36 | 10.28 | 9.81 |
| *Firmicutes* | 6.94 | 15.82 | 11.57 | 4.41 |
| *Acidobacteria* | 6.82 | 6.25 | 6.23 | 4.88 |
| *Verrucomicrobia* | 2.67 | 2.15 | 3.09 | 3.26 |
| *Chloroflexi* | 2.47 | 2.50 | 2.23 | 2.21 |
| *Cyanobacteria* | 1.55 | 0.91 | 1.32 | 2.33 |
| *Planctomycetes* | 1.38 | 1.18 | 1.23 | 1.01 |
| *Gemmatimonadetes* | 0.59 | 0.60 | 0.46 | 0.41 |
