# Supplementary material for: Effects of Water Loss Stress under Tidal Effects on the Epiphytic Bacterial Community of Sargassum thunbergii in the Intertidal Zone
Source: mSphere. 2022 Sep 29;7(5):e00307-22. doi: 10.1128/msphere.00307-22 (PMC9599519; doi:10.1128/msphere.00307-22)
Supplement: TABLE S5 [file msphere.00307-22-s0006.docx]

| Genus | H0 | H2 | H4 | H5 |
| --- | --- | --- | --- | --- |
| *uncultured_bacterium_f_Saprospiraceae* | 10.63 | 11.65 | 7.86 | 9.01 |
| *uncultured_bacterium_f_Microtrichaceae* | 6.09 | 5.44 | 4.81 | 4.05 |
| *uncultured_bacterium_c_Gammaproteobacteria* | 3.54 | 7.64 | 3.55 | 5.17 |
| *Granulosicoccus* | 6.00 | 4.54 | 4.62 | 4.09 |
| *uncultured_bacterium_f_Rhodobacteraceae* | 3.79 | 4.15 | 4.08 | 3.99 |
| *Acinetobacter* | 2.31 | 2.42 | 4.60 | 1.43 |
| *Portibacter* | 1.68 | 2.23 | 2.15 | 4.21 |
| *Exiguobacterium* | 1.44 | 5.72 | 2.36 | 0.35 |
| *Sva0996_marine_group* | 2.95 | 1.83 | 2.26 | 2.12 |
| *Aquimarina* | 1.01 | 3.47 | 0.87 | 2.22 |
| Genus | M0 | M2 | M4 | M5 |
| *uncultured_bacterium_f_Saprospiraceae* | 12.43 | 17.46 | 6.21 | 5.67 |
| *uncultured_bacterium_f_Microtrichaceae* | 5.41 | 6.69 | 4.10 | 3.32 |
| *uncultured_bacterium_f_Rhodobacteraceae* | 5.04 | 5.62 | 4.48 | 2.90 |
| *uncultured_bacterium_c_Gammaproteobacteria* | 2.31 | 3.79 | 4.53 | 5.20 |
| *Granulosicoccus* | 3.75 | 3.73 | 4.30 | 4.05 |
| *Portibacter* | 1.48 | 2.61 | 1.79 | 3.61 |
| *Acinetobacter* | 4.20 | 1.62 | 0.49 | 1.55 |
| *Sva0996_marine_group* | 2.52 | 2.18 | 1.44 | 1.09 |
| *Aquimarina* | 0.25 | 5.06 | 0.98 | 0.62 |
| *uncultured_bacterium_o_Subgroup_2* | 1.66 | 1.11 | 2.29 | 1.38 |
| Genus | F0 | F2 | F4 | F5 |
| *uncultured_bacterium_f_Saprospiraceae* | 8.95 | 5.56 | 9.36 | 12.12 |
| *uncultured_bacterium_c_Gammaproteobacteria* | 4.68 | 11.68 | 2.66 | 5.14 |
| *Granulosicoccus* | 8.10 | 5.40 | 4.92 | 4.13 |
| *uncultured_bacterium_f_Microtrichaceae* | 6.73 | 4.14 | 5.46 | 4.73 |
| *Exiguobacterium* | 2.43 | 11.12 | 4.43 | 0.05 |
| *uncultured_bacterium_f_Rhodobacteraceae* | 2.62 | 2.61 | 3.71 | 5.01 |
| *Acinetobacter* | 0.56 | 3.26 | 8.34 | 1.31 |
| *Portibacter* | 1.87 | 1.84 | 2.48 | 4.77 |
| *Sva0996_marine_group* | 3.34 | 1.47 | 3.02 | 3.08 |
| *Aquimarina* | 1.72 | 1.81 | 0.76 | 3.72 |
